# Diagnostic and Therapeutic Perspectives Associated to Cobalamin-Dependent Metabolism and Transcobalamins’ Synthesis in Solid Cancers

**DOI:** 10.3390/nu14102058

**Published:** 2022-05-14

**Authors:** Valentin Lacombe, Guy Lenaers, Geoffrey Urbanski

**Affiliations:** 1 MitoLab Team, MitoVasc Institut, CNRS UMR6015, INSERM U1083, Angers University, 49000 Angers, France; guy.lenaers@inserm.fr (G.L.); urbanskigeoffrey@gmail.com (G.U.); 2 Department of Internal Medicine and Clinical Immunology, Angers University Hospital, 49000 Angers, France; 3 Department of Neurology, Angers University Hospital, 49000 Angers, France

**Keywords:** neoplasms, vitamin B12, transcobalamins, methionine, metabolism, methylation

## Abstract

Cobalamin or vitamin B12 (B12) is a cofactor for methionine synthase and methylmalonyl-CoA mutase, two enzymes implicated in key pathways for cell proliferation: methylation, purine synthesis, succinylation and ATP production. Ensuring these functions in cancer cells therefore requires important cobalamin needs and its uptake through the transcobalamin II receptor (TCII-R). Thus, both the TCII-R and the cobalamin-dependent metabolic pathways constitute promising therapeutic targets to inhibit cancer development. However, the link between cobalamin and solid cancers is not limited to cellular metabolism, as it also involves the circulating transcobalamins I and II (TCI or haptocorrin and TCII) carrier proteins, encoded by *TCN1* and *TCN2*, respectively. In this respect, elevations of B12, TCI and TCII concentrations in plasma are associated with cancer onset and relapse, and with the presence of metastases and worse prognosis. In addition, *TCN1* and *TCN2* overexpressions are associated with chemoresistance and a proliferative phenotype, respectively. Here we review the involvement of cobalamin and transcobalamins in cancer diagnosis and prognosis, and as potential therapeutic targets. We further detail the relationship between cobalamin-dependent metabolic pathways in cancer cells and the transcobalamins’ abundancies in plasma and tumors, to ultimately hypothesize screening and therapeutic strategies linking these aspects.

## 1. Introduction

The identification of metabolic pathways promoting cancer cell growth is of major interest in oncology [1,2]. Cobalamin, or vitamin B12 (B12), is essential for cell proliferation [3]; consequently, cobalamin-dependent pathways are of high interest to target cancer cells. Cobalamin is a cofactor for two enzymes: methionine synthase and methylmalonyl-CoenzymeA mutase (MMCoAMut), which are implicated in methylation, purine bases synthesis, succinylation and ATP production [3]. These functions are crucial within tumor cells for their proliferation, explaining why the avidity of tumor cells for cobalamin is crucial [4], while the inhibition of B12 uptake in vitro has anti-proliferating effects [5].

In addition to the high B12 needs and uptake by cancer cells, the link between cobalamin and neoplasms also involves B12-binding proteins, the transcobalamins. Before the identification of transcobalamins in the 1960s, it was demonstrated that the plasma cobalamin-binding capacity was raised in myeloid blood malignancies (not detailed in this review) and in solid cancers [6,7], paralleling the accumulation of a yet unknown cobalamin-binding protein [7]. A few years later, the identification and dosage of transcobalamins [8,9] led to the observation of high transcobalamin levels in solid cancers [10,11,12]. The elevation of total plasma B12 (tB12) and plasma transcobalamins was further associated with solid cancer diagnosis [13,14], and also with metastases [15] and a worse clinical prognosis [16].

Today, cobalamin and transcobalamins can be considered in solid cancers either as biomarkers of the diagnosis [15,17,18,19] or the prognosis of cancers [16], or as therapeutic targets to affect the avidity of cancer cells for cobalamin [5,20,21,22] or the cobalamin-dependent metabolic pathways [23,24,25,26]. However, previous reports focused either on plasma tB12 or transcobalamins as biomarkers in clinical research studies, or on the cobalamin-dependent pathways and transcobalamin synthesis in basic research studies, but never both together. Thus far, no hypothesis linking these two aspects has been suggested. In this review, we detailed the relationship between the cobalamin-dependent metabolic pathways, the tumor or plasma transcobalamins levels, and their implications in cancer cell growth to formulate relevant hypotheses for future diagnostic and therapeutic perspectives.

## 2. Cobalamin: From Absorption to Intracellular Metabolism

Cobalamin is only produced by certain bacteria and archaea, and is composed of a corrin ring centered by a cobalt atom in mono, bi or trivalent form, which links a variable residue by either adenosyl-, methyl-, hydroxyl- or cyano- group [27]. Cobalamin is an essential vitamin for human beings as human cells cannot synthetize it, and its daily dose comes from ruminants and fish meat as the main food source [3,28].

Cobalamin is released from food cobalamin-binding proteins by the acidity of gastric juice, then carried by salivary haptocorrin, which is later degraded by pancreatic enzymes. Then, cobalamin is linked to the intrinsic factor produced by gastric parietal cells and uptaken via endocytosis by the cubam receptor, a complex formed by the cubulin and the amnionless transmembrane protein, into the terminal ileum [3]. A small cobalamin part is also absorbed via passive diffusion throughout the intestine [29,30]. Once internalized into the enterocytes, the cobalamin is transferred into the lysosome and then transported into the blood [31].

**Figure 1:**
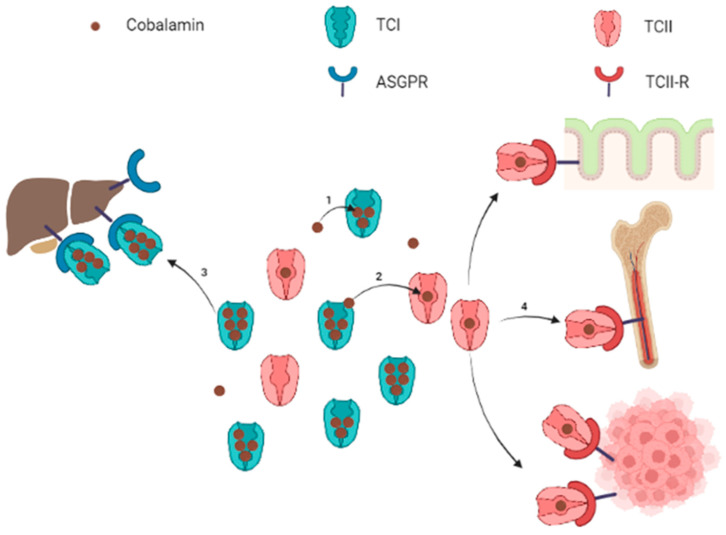
Schematic representation of circulating cobalamin bound to transcobalamins and uptake of transcobalamin-bound cobalamin by target organs. Note: Free cobalamin represents a small part of the total circulating cobalamin. Cobalamin is mostly carried by TCI (1), which constitutes a circulating pool of quickly available cobalamin. Cobalamin carried by TCI can be either transferred to TCII (2) or internalized into liver cells for storage throughout the ASGPR (3). The TCII-bound cobalamin is picked up by cells expressing the TCII-R when cobalamin is required for metabolism (4). The TCII-R is particularly expressed by epithelial cells, bone marrow and in physiological or pathological highly proliferating cells. ASGPR: asialoglycoprotein receptor; TCI: transcobalamin I (haptocorrin); TCII: transcobalamin II; TCII-R: transcobalamin II receptor. Figure created with BioRender.com accessed on 25 April 2022.

Circulating cobalamin is carried by two proteins: transcobalamin I (TCI, also called haptocorrin) and transcobalamin II (TCII, Figure 1). TCI belongs to R-binder proteins and is encoded by the *TCN1* gene. TCI is present in various body fluids and is secreted by many cell types including glandular cells and granulocytes [32,33]. TCI carries most of the blood circulating cobalamin [31,34,35]. Salivary TCI is also important for the enteral cobalamin absorption, and plasma TCI allows liver storage of cobalamin via the asialoglycoprotein receptor (ASGPR) [36]. The ASGPR is highly present in the hepatocytes’ membrane, but the ASGPR has also been found with a lower expression in other normal tissues (salivary glands, small intestine, testes, thyroid, kidneys, brain, lung) [4,37,38,39]. Hepatocellular carcinoma cells have variable levels of ASGPR expression [40,41], but to our knowledge, no study has demonstrated the absorption of TCI-bound cobalamin through the ASGPR in non-liver malignant cells. Quadros E. suggested specific roles for TCI: the large proportion of cobalamin bound to TCI prevents the loss of free Cbl, and the relatively low specificity of TCI for cobalamin allows TCI to link corrin-like compounds, called corrinoids, to limit their cell uptake for metabolic use via the TCII-receptor pathway [33].

TCII is encoded by the *TCN2* gene and binds 10 to 30% of the plasma cobalamin [31,33,34,35]. Circulating TCII is mainly synthetized by endothelial cells, although many kinds of cells may produce faint TCII levels [33,42,43,44,45,46]. TCII is highly specific for cobalamin and is more selective, binding cobalamin rather than corrinoids, compared to TCI. The cobalamin-TCII complex, called holotranscobalamin, binds to the membrane TCII-receptor (TCII-R) in order to provide cobalamin to all cells. The expression of TCII-R on the cell surface is increased in actively dividing cells, whereas it is decreased in quiescent cells [47]. This regulation combined to the efflux of a cellular excess of cobalamin promotes its availability to cells most needing it.

Inside cells, the cobalamin-TCII complex is dissociated in the lysosome and TCII-R is recycled to the plasma membrane, while cobalamin is released into the cytoplasm [33]. Ultimately, cytoplasmic cobalamin either remains in the cytoplasm or is transferred into the mitochondria to form methylcobalamin or adenosylcobalamin, respectively [3].

**Figure 2:**
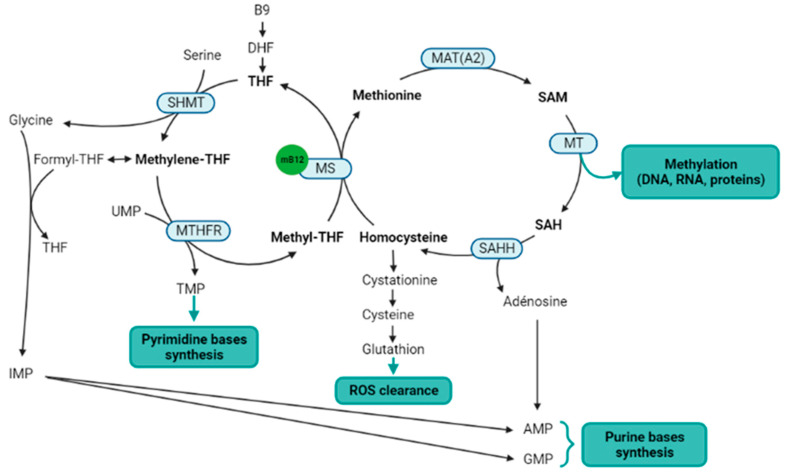
Metabolic pathways directly or indirectly related to the methylcobalamin. Notes: Methylcobalamin is a cofactor for methionine synthase, which is the central enzyme of the one-carbon metabolism (metabolites in bold) that includes the folate cycle (**left**) and the methionine cycle (**right**). Two methionine salvage pathways are not represented in this figure: a first salvage pathway allows methionine synthesis from homocysteine and betaine thanks to the cobalamin independent betaine-homocysteine methyltransferase, and a second salvage pathway allows the methionine synthesis from SAM via the polyamine pathway. B9: vitamin B9; AMP: adenosine monophosphate; DHF: dihydrofolate; GMP: guanosine monophosphate; IMP: inosine monophosphate; MAT(A2): methionine adenosyltransferase (notably MATA2); mB12: methylcobalamin; MS: methionine synthase; MT: methyltransferases; MTHFR: methylenetetrahydrofolate reductase; SAH: S-adenosylhomocysteine; SAHH: SAH hydrolase; SAM: S-adenosylmethionine; THF: tetrahydrofolate; TMP: thymidine monophosphate; UMP: uridine monophosphate. Figure created with BioRender.com accessed on 25 April 2022.

Methylcobalamin is a cofactor for the methionine synthase, which catalyzes the methyl transfer from 5-methyltetrahydrofolate to homocysteine, to produce methionine and tetrahydrofolate [48]. Methionine synthase is a key enzyme of the one-carbon metabolism and methylation process, straddling the folate and methionine cycles (Figure 2). Indeed, methionine is thereafter transformed into S-adenosylmethionine (SAM), which is the universal methyl donor by transformation into S-adenosylhomocysteine (SAH) [49]. Methylcobalamin and methionine synthase are therefore directly involved in the methylation process. Salvage methionine synthesis pathways exist independently of folate and cobalamin, based on the polyamine pathway and the betaine-homocysteine methyltransferase [50], but these pathways are of minor importance and the inhibition of the methionine synthase results in a severe decrease in methylation reactions [51]. In addition, the methionine cycle is involved in reactive oxygen species (ROS) clearance via the transsulfuration pathway, and SAM is also itself an allosteric activator of the transsulfuration pathway through the cystathionine beta synthase (CBS). The activation of CBS is associated with the production of H2S to stimulate angiogenesis [52,53]. Finally, the methionine cycle is also involved in purine bases synthesis through the adenosine generation from SAH.

**Figure 3:**
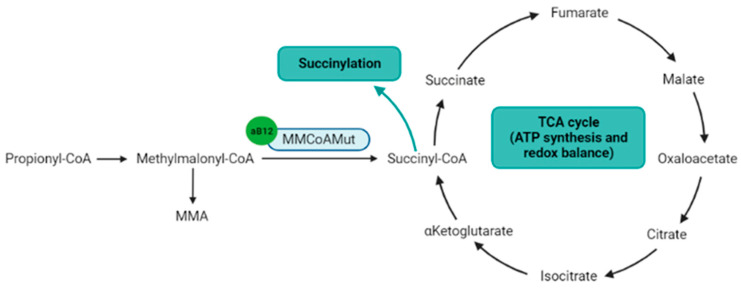
Metabolic pathways directly or indirectly related to the adenosylcobalamin. Notes: Adenosylcobalamin is a cofactor for MMCoAMut enzyme that synthesizes succinyl-CoA, which is a component of the TCA cycle and the substrate for lysine succinylation. aB12: adenosylcobalamin; MMA: methylmalonic acid; MMCoAMut: methylmalonyl-CoA mutase; TCA cycle: tricarboxylic acid cycle (Krebs cycle). Figure created with BioRender.com accessed on 25 April 2022.

Adenosylcobalamin is a cofactor for the methylmalonyl-CoenzymeA mutase (MMCoAMut), which converts methylmalonyl-CoA to succinyl-CoA (Figure 3) [54]. Succinyl-CoA is involved in the succinylation processes, and is a major component of the tricarboxylic acid (TCA) Krebs cycle, which produces ATP, NADH and FADH2, the latter two to feed the respiratory chain to produce ATP [55]. Therefore, the MMCoAMut activity on one hand feeds the TCA cycle, and on the other hand contributes to lysine succinylation and downstream-associated posttranslational modifications [56].

## 3. Relationship between Cobalamin, Transcobalamins and TCII-R in the Context of Solid Cancer

### 3.1. Measurements of Plasma Cobalamin and Transcobalamins in Clinical Practice

Evaluating the total serum or plasma (tB12) concentration is usually aimed at detecting a deficiency, but an incidental finding of elevated tB12 is not uncommon [15,57]. This latter situation should prompt the search for specific medical conditions, such as myeloid blood malignancies, liver diseases, auto-immune diseases, chronic tubulointerstitial nephritis and severe renal failure [10,17,58]. In current medical practice, tB12 is measured without distinction between the free cobalamin, the holohaptocorrin (cobalamin linked to TCI, B12-TCI), which represents the main part of plasma cobalamin, and the holotranscobalamin (cobalamin linked to TCII, B12-TCII), which is involved in delivering cobalamin to cells. In healthy subjects with a physiological tB12 concentration, a correlation between tB12, B12-TCI and B12-TCII has been described [59,60]. However, these results cannot be extrapolated to patients with elevated tB12, potentially due to the increased concentrations of TCI, TCII or both.

### 3.2. Association between tB12, TCI, TCII and the Diagnosis of Solid Cancers

In the 1970s, very high levels of tB12, TCI and TCII were reported in patients with solid cancers and without any other elevated B12-related causes [10,58,61]. Since these first descriptions, most studies have focused on tB12m which is a common dosage in clinical practice. Consequently, tB12 level was found to be higher at the diagnosis of esophagus, stomach and liver cancers, than in healthy subjects [62]. Recently, two studies confirmed the association between solid cancers and elevated plasma tB12 in large registries. In a Danish registry, plasma tB12 levels >800 pmol/L (1084 ng/L) were associated with solid cancer diagnosis, with a standardized incidence ratio of 6.3 [95% CI: 5.7–6.9] [13]. Similarly, in a British registry, plasma tB12 levels >1000 pmol/L (>1355 ng/L) were associated with solid cancer diagnosis during the following year after the tB12 measurement, with an incidence rate ratio of 4.7 [95% CI: 4.0–5.6] [14]. In a case-control study, we confirmed these results after an adjustment for each of the other elevated B12-related causes, showing that tB12 level ≥1000 ng/L was associated with solid cancer without metastasis (OR 2.0 [95% CI: 1.2–3.3]) or with metastasis (OR 4.2 [95% CI: 2.7–6.6]) [15].

Elevated plasma B12 was clearly associated to primary cancer sites from the colorectum, stomach, esophagus, lung, pancreas and urothelium, while data were discordant for liver and prostate cancers [13,14,15,63,64]. The association between liver cancer and elevated B12 level was debated, with conclusions often differing according to the adjustment, or not, to confounding factors. In this respect, the association between hepatocellular carcinoma and plasma B12 elevation is related to the underlying chronic liver disease [15,65]. Focusing on lung cancers, an association between elevated levels of plasma TCI and adenocarcinoma was disclosed, whereas no association with squamous cell carcinoma was found [66], highlighting the importance of considering the histological type of cancers, rather than the cancer sites, for the association with the elevated plasma tB12 or TCI.

Nevertheless, the interest in plasma tB12 measurement for cancer diagnosis remains a frequent matter of debates [13,14,15,18,19]. Indeed, elevated tB12 levels were fortuitously found to be associated to many other diseases, which complicated the interpretation of this result in a cancer screening strategy. In addition, there are no guidelines to help explorations in the case of high B12 discovery, in particular for the diagnosis of solid cancer [18,19,57]. However, a consensus emerged to first eliminate the frequent causes associated with elevated plasma tB12, mainly myeloid blood malignancies and liver diseases, before assessing the presence of a solid cancer. In a retrospective study, we demonstrated that the plasma tB12 elevation should be controlled before searching for a cancer in the absence of a clinical sign [19]. Indeed, a transient tB12 elevation was not associated with cancer diagnosis. On the contrary, a persistent elevated tB12 level, defined by two tB12 measurements ≥1000 ng/L at least four weeks apart, led, within the next five years, to a diagnosis of incident solid cancer in 20.8% of patients, versus 3.8% in the case of normal B12 measurements and 6.0% in the case of transient B12 elevation (*p* < 0.001). Confirming the tB12 elevation could therefore avoid useless worrying for the patient and expensive investigations. The association between elevated plasma transcobalamin levels and solid cancers questions their relevance and performance in a cancer diagnosis strategy. However, measurements of transcobalamins are not performed routinely, and no prospective study has yet assessed the interest of TCI or TCII measurements to diagnose cancers.

### 3.3. Association between tB12, TCI, TCII and the Prognosis of Solid Cancers

The first observations of elevated tB12 or transcobalamins in solid cancers were reported to be associated to a bad prognosis [10,58]. In a population-based cohort study, a lower one-year survival rate was observed in individuals with elevated plasma tB12: 35.8% [95% CI: 33.2–38.4] when the plasma tB12 > 800 pmol/L (1084 ng/L) versus 69.3% [95% CI: 68.7–70.0] in control cases with a normal tB12 level (200–600 pmol/L, *p* < 0.001) [16]. We further showed that the presence of metastases increased the association between solid cancer and elevated plasma tB12 (>1000 ng/L), suggesting a link between the elevation of plasma B12, the tumor mass and its proliferative capacity [15]. The survival of patients with cancer was prospectively assessed in another study, demonstrating that high levels of tB12 and C-reactive protein (CRP) are two independent biomarkers for a worse prognosis [67]. In this study dedicated to patients in palliative care for solid cancers, the survival rate was inversely associated with the tB12 levels: 85% of patients with a tB12 level > 600 pmol/L (813 ng/L) died in the first three months, versus 57% in patients with a tB12 level <300 pmol/L (406 ng/L).

### 3.4. Association between tB12, TCI, TCII Changes and the Course of Solid Cancers

In a small retrospective study including patients with elevated tB12 and solid cancers, we demonstrated that plasma tB12 increased in cancer patients with supportive care (+157.4 ng/L/month), while it decreased in patients with curative care (−171.6 ng/L/month, *p* = 0.001) [68]. Such findings were also described in case reports and small series assessing the evolution of TCI or TCII levels during the cancer course while denoting an elevation at diagnosis, followed by a normalization after a curative treatment, and eventually a new increase during relapses [11,69,70,71,72,73]. These data are in line with a study demonstrating that TCI synthesis decreases in colorectal cancer cells after chemotherapy [74]. More recently, elevated plasma TCII appeared to be a good indicator of disease progression in a prospective study involving 20 patients with metastatic renal adenocarcinoma [70]. All these findings argue for a parallel evolution of the plasma tB12, TCI and TCII levels and the cancer course, and questions the interest in repeating these measurements in a prospective study during patients’ follow-up, for the early detection of a lack of treatment response or relapse.

### 3.5. Direction of the Causal Link between Plasma tB12, TCI or TCII Measurements and the Presence of Solid Cancers

The demonstration that elevated levels of plasma tB12, TCI and TCII are associated with solid cancers questions the causal link of this association: are the elevated concentrations of tB12, TCI and TCII causing the development of solid cancer or are they the consequences of an underlying cancer? In the cohorts’ follow-up, the diagnosis of cancer was more frequent during the first year following B12 measurement [13,14,19]. This short-term association suggests that B12 elevation is related to an underlying and still undiagnosed infraclinical cancer. Nevertheless, few authors have supported that the elevated B12 could be a condition favoring cancer onset and its progression [75,76]. The recent demonstration of a tB12 normalization after curative treatment in patients with elevated B12 at the time of the cancer diagnosis strongly argues for considering that tB12 elevation is secondary to solid cancers [68,77]. Supporting this, basic research demonstrated that tumor tissue samples from patients with elevated plasma transcobalamins contain higher concentrations of TCI and TCII than control tissues [78]. Similar conclusions were drawn after demonstrating that patients with gastric cancer had higher plasma TCI levels than those with benign gastric pathology, and that TCI production was significantly higher in gastric tumor samples than in healthy gastric mucosa samples [77]. These data strongly argue for a secretion of transcobalamins by cancer cells or by their microenvironment.

### 3.6. Cobalamin Avidity and TCII-R Expression in Cancer Cells

Many data suggest an important role for TCII-R in cobalamin uptake in the situation of high cellular anabolism: (i) corticosteroid treatment raises the expression and activity of TCII-R [79], (ii) mitogen stimuli raise the TCII-R expression in lymphocytes [80], (iii) cancer cells have an elevation in cobalamin uptake [4], and (iv) the expression of TCII-R is upregulated in several cancers [81]. The high expression of TCII-R in solid cancer cells was demonstrated using TCII-R immune-histological assessments and *CD320* RT-qPCR analyses [81,82], and using radiolabeled cobalamin or analogs to label tumors [4,22,83,84,85,86], suggesting that the level of cobalamin uptake is associated to cancer aggressiveness [4].

The cobalamin uptake by the TCII/TCII-R pathway is crucial for cancer cell proliferation. Indeed, anti-TCII-R antibodies [5] and TCII-R downregulation by siRNA inhibit cobalamin uptake and the proliferation of malignant cells in vitro [87]. The expression of the TCII-R rises in proliferating cells and decreases in quiescent cells [47,88,89], paralleling TCII secretion levels in cells with high proliferating capacity and short doubling time [44,90]. Immunohistochemistry quantification of TCII, TCII-R and Ki-67 expressions in 34 human xenografts from various tumor types [81], demonstrated that all stained positively for TCII and TCII-R, highlighting the importance of the TCII/TCII-R pathway in solid cancers. Nevertheless, no association was observed with Ki-67 expression, conversely to the hypothesis supporting a strict link with the proliferating capacity.

Comparative studies of methionine-dependent and methionine-independent cells derived from the human glioma cell line GaMg, demonstrated that incubation into a homocysteine-rich medium depleted of methionine is associated with increased TCII-R expression [91], which was much more marked in the methionine-dependent cell line, and associated to increased levels of intracellular cobalamin content. This work demonstrated that the cellular avidity for cobalamin correlates to the needs of cobalamin-dependent methionine biosynthesis.

### 3.7. TCN1 and TCN2 Gene Mutations and Expression in Cancer Cells

Analyses of the gene microarray datasets between colorectal tumors and normal colorectal tissues identified a tumor transcriptional signature [92], among which, *TCN1* was one of the eight genes most expressed among the 14,698 genes considered. Similarly, *TCN1* upregulation was identified as one of most relevant predictive marker of a poor response to chemotherapy [74,93]. This was further confirmed by *TCN1* immunohistochemistry analyses disclosing that high *TCN1* expression is predictive of a worse disease-specific mortality (HR 3.3 [95% CI: 1.6–7.1], *p* = 0.002), a worse relapse rate (HR 3.0 [95% CI: 1.1–8.7], *p* = 0.04) and a worse metastasis-free survival (HR 3.0 [95% CI: 1.2–7.7], *p* = 0.02) [93]. Such findings related to cytoplasmic TCI and mRNA *TCN1* overexpressions were also demonstrated for the locally advanced hypopharyngeal squamous cell carcinoma [94], with a lower response to neoadjuvant chemotherapy. Conversely, *TCN1* silencing suppressed cell growth and increased cisplatin sensitivity of the FaDu hypopharyngeal squamous carcinoma cell line [94].

In addition, *TCN1* and *TCN2* single nucleotide polymorphisms (SNP) were associated with the risk of digestive cancer onset. The *TCN1* intronic SNP rs526934 was associated with gastric cancer risk (OR 2.09 [95% CI: 1.25–3.51]) [75] and the risk of colorectal cancer was increased in homozygote subjects with the *TCN2* c.776C>G (OR 2.9 [95% CI: 1.1–7.6]) and the c.1026-394T>G (OR 3.1 [95% CI: 1.2–8.2]) variant alleles [95].

Furthermore, *TCN2* downregulation in glioblastoma cell lines decreased cobalamin intracellular concentration in hypoxic conditions, stimulating the epithelial–mesenchymal transition (EMT) process, and promoting migratory and invasive properties, and cancer stem cell (CSC) differentiation [96]. Indeed, *TCN2* repression leads to a phenotype similar to the one induced by hypoxic conditions with EMT and CSC transformations, whereas *TCN2* overexpression inhibits EMT and CSC processes. Thus, *TCN2* regulation has an important role in dictating the phenotype of the cancer cell.

## 4. Implication of Cobalamin-Dependent Pathways in Tumor Initiation and Cell Proliferation

### 4.1. Methionine Synthesis and Methylation

Being a cofactor for methionine synthase, cobalamin is involved in methionine synthesis, which is crucial for methylation processes and is also related to ROS scavenging. Methionine is a sulfur-containing essential amino acid with an unbranched flexible side chain that confers a malleable surface to proteins rich in methionine [97]. The methionine’s sulfur-aromatic motif stabilizes protein structures and interactions between proteins [98]. In addition, methionine residues confer an antioxidant function, scavenging ROS without any deleterious effect on most protein activities [99]. Nevertheless, for some proteins, methionine sulfoxidation may regulate processes such as the inhibition of transcription factors degradation [97,100] or the control of kinase and phosphatase activities [97]. Finally, methionine is, above all, the precursor of SAM, which is the universal methyl donor used by methyltransferases for the methylation processes.

Methionine is also a singular amino acid because, comparatively to all other amino acids, methionine deprivation induces a dramatic transcriptional response involving the upregulation and downregulation of a large panel of genes, mediated mainly by the reduction in histone methylation [101]. For example, methionine restriction activates the *Hoxa5* homeobox gene, encoding a transcription factor important for tumorigenesis [102,103], as *Hoxa5* abundance correlates with p53 expression [104], and its downregulation parallels the transition from normal colon tissue to adenoma, then carcinoma [105]. In addition, low *Hoxa5* expression is associated with tumor-node-metastasis (TNM) stages, tumor size and poor prognosis in non-small cell lung cancer, by controlling cell proliferation via the positive regulation of the *Cdkn1a* expression [106], whereas increasing HOXA5-dependent genes explains the anti-tumor effect of methionine deprivation [101].

DNA, RNA and protein methylations are one of the main epigenetic pathways regulating gene expression, RNA translation and protein function and interaction [49,107,108]. DNA methylation within the gene corpus leads to transcription activation, whereas DNA methylation of CpG islands from promotors may lead to gene repression [109,110]. Thus, according to the context and distribution of the DNA methylation sites, methylation will act as a positive or negative switch [111,112]. In cancer cells, hypomethylation is frequently observed [113], although 5 to 10% of physiologically unmethylated CpG islands become abnormally highly-methylated [111]. This can affect the expression of noncoding RNAs involved in oncogenesis [112]. Finally, DNA methylation is an epigenetic process implicated in the silencing of tumor suppressor genes that favor the tumor initiation and progression [114]. Histones’ methylation also influences gene transcription by modulating the chromatin condensation to regulate the access to transcription factors. These processes repress transcription in cases of histone methylation on lysines H3K9, H3K27 or H4K20, and favor transcription in cases of H3K4 methylation. Histone demethylation is implicated in the differentiation of normal cells and tissues, by favoring the transcription of specific genes [115,116,117], while histone methylation is associated with dedifferentiation and aggressiveness in cancer [118,119].

There are different reasons to explain cancer cells’ dependency to methionine [120,121,122]. First, the intense cell proliferation increases methionine needs to ensure protein synthesis and the methylation process. Second, some cancers lack methylthioadenosine phosphorylase (MTAP), an enzyme allowing the synthesis of methionine from methylthioadenosine by the polyamine pathway, either because the MTAP gene is methylated or co-deleted with the tumor suppressor gene p16 [123]. Third, some tumors have a low level of methionine synthase, prompting a dependency on exogenous methionine intake [24]. Consequently, many cell lines are unable to grow in a medium containing homocysteine without methionine, contrarily to normal cells [121,124,125], due to a cell cycle arrest before mitosis related to the inhibition of cyclin-dependent kinases CDK2 and cdc2 [126,127].

The importance of the one-carbon cycle in tumor-initiating cells from lung adenocarcinoma was demonstrated by observing elevated one-carbon cycle activity and transmethylation rates, with high levels of methionine consumption, mandatory for driving the tumorigenesis [128]. Indeed, a 48-h methionine deprivation leads to a major reduction in SAM, and a decrease in histones methylation and cell growth that cannot be reversed by secondary methionine addition in the culture medium. Conversely, temporary restrictions in various other amino acids do not impact to the same extent the tumorigenesis and cell growth. The importance of the one-carbon cycle in tumorigenesis and tumor growth also requires other enzymes and compounds related to this cycle, for promoting its functions [129,130]. For example, high levels of enzymes such as SHMT2, MTHFD2 and ALDH1L2, which are involved in the folate cycle part of the one-carbon metabolism, are observed in colic cancers and associated with their prognosis [131].

### 4.2. Succinylation

Being a cofactor for MMCoAMut, cobalamin in involved in succinyl-CoA synthesis and therefore in succinylation. Lysine succinylation has been identified as an important posttranslational modification of proteins [132], converting the cationic to anionic side chain of this amino acid, leading to modifications of protein charges and structures [133]. For example, histone hyper-succinylation is correlated with active gene expression in cancer initiation and growth [134]. Various enzymes exhibit a high succinyltransferase activity, such as KAT2A (lysine acetyltransferase 2A) that succinylates H3K79, with a maximal affinity for gene transcription start sites, promoting tumor growth [135]. Similarly, HAT1 (histone acetyltransferase 1), which succinylates H3K122, contributing to epigenetic gene regulation in cancer cells and PGAM1 (Phosphoglycerate Mutase 1) in a non-histone region, stimulates glycolytic fluxes in cancer cells [136]. Additionally, CPT1A (carnitine palmitoyltransferase 1A) has a lysine succinyltransferase activity to promote S100A10 succinylation, supporting gastric cancer progression [137]. Indeed, S100 proteins are a family of calcium-binding cytosolic proteins with important roles in the invasion and migration phases of tumorigenesis. Finally, succinylation of PKM2 (pyruvate kinase M2) under glucose starvation conditions plays a role in the metabolism switch from proliferation to cell survival, and vice versa in colon cancer, leading to cellular survival under stressful conditions [138].

### 4.3. Other Functions Indirectly Related to the Cobalamin-Dependent Enzymes

*Purine bases’ synthesis.* Intracellular synthesis of purine bases is essential in cancer to ensure DNA replication [49]. By contributing to the adenosine synthesis via the SAH, the one-carbon cycle, and therefore the cobalamin-dependent methionine synthase, is involved in purine bases’ synthesis.

*Reactive oxygen species regulation.* The relationship between ROS and cancer progression or treatment is complex [139]. The activation of antioxidative pathways confers radio-resistance [140,141], and the generation of ROS by drugs such as cisplatin, is used for cancers’ treatment to induce a cytotoxicity through DNA damages [142]. Thus, the ability of cancer cells to clear ROS contributes to chemo- and radio-resistance processes [139]. The cobalamin-dependent methionine synthase is important for ROS clearance because methionine residues act as ROS scavengers [97,99], and because the transsulfuration pathway derived from the methylation cycle results in the production of glutathione, also involved in ROS clearance [143].

*ATP production and redox regulation* via *the TCA cycle.* MMCoAMut, for which cobalamin is a cofactor, is the provider of succinyl-CoA an intermediate of the tricarboxylic (TCA) cycle, which is important in cancer cells because it generates cofactors for redox reactions, allowing mitochondrial ATP synthesis [143], a process essential for cancer initiation and growth [55,144]. The TCA cycle is also a source of biosynthetic chemical intermediates such as α-ketoglutarate and 2-hydroxyglutarate that regulate histone and DNA methylation [133,145]. Importantly, pathogenic variants in three genes encoding TCA enzymes, succinate dehydrogenase [146], fumarate hydratase [147] and isocitrate dehydrogenase [148], are associated to increased cancer risk, providing further arguments linking the altered TCA cycle to tumorigenesis [149].

## 5. Potential Therapeutic Uses Derived from Cobalamin Avidity and Methionine Needs of Cancer Cells

### 5.1. Using Cobalamin as a Vector for a Trojan Horse Effect

Due to their high cobalamin avidity, expression of TCII-R constitutes a potential vehicle for the import of antineoplastic drugs into cancer cells [22]. For example, cobalamin was used as a Trojan horse by administering nitrosylcobalamin, a vitamin B12-based non-toxic carrier of nitric oxide (NO), to release toxic NO in cancer cells [21]. Additionally, paclitaxel-loaded cobalamin-containing micelles were developed to counteract paclitaxel resistance of gastric cancer due to drug-efflux pumping. This process promotes the access of paclitaxel to the cancer site and minimizes the severe cytotoxic collateral side effects of this drug [150]. As a possible treatment, these micelles enhanced cellular uptake, reversed drug resistance in vitro and in vivo, and were well tolerated in vivo. More recently, the development and use of cobalamin-labeled nanoparticles containing miR-532-3p were shown to induce apoptosis of gastric cancer cells [151]. These examples illustrate different ways to use cobalamin avidity to target cancer cells as a Trojan horse.

### 5.2. Inhibition of the One-Carbon Cycle

*Methionine restriction*. Methionine restriction is a strategy consisting of depriving cancer cells of exogenous methionine. A low methionine diet leads to decreased purine bases and ATP syntheses, and decreased DNA and histone methylations, with drastic consequences on demethylated gene transcription [49,152]. Many studies have documented the benefits of methionine restriction and depletion associated to methioninase, to inhibit tumor growth [23,24,25,120,153]. This was first demonstrated in rats in 1959 [154]. Subsequent studies confirmed the efficacy of oral [155], parenteral [156] and intravenous methionine-free diets [157], or enzymatic depletion of methionine by oral methioninase treatment [23,153], alone or associated with different chemotherapy protocols. The effect of methioninase treatment on methionine plasma levels is more pronounced compared to methionine-free diets, with an expected better efficiency against cancer evolution [153,158]. Importantly, these treatments are well tolerated because normal cells are able to synthetize a sufficient methionine pool from homocysteine to meet their own needs during the treatment period, contrary to cancer cells, whose needs are higher for the previously described reasons. The anti-tumor effects of methionine deprivation have been studied in various cancer types in experimental studies on animal and in pilot human studies [24,25,120,158], but only one phase II clinical trial was performed with a large study population [157]. This latter study included 138 patients with advanced gastric cancers, and compared a 14 days total parenteral nutrition with a methionine-free amino acid solution, to a commercial amino acid solution, by intravenous administration. Results demonstrated that methionine restriction significantly potentiated the effect of chemotherapy by 5-fluorouracil and mitomycin C, with a significant partial or complete increase in clinical response rates in the methionine-depleted group (26.3% versus 8.1%, *p* = 0.015) [157], and of histological responses among patients with gastrectomy (*p* = 0.016) [159], without a difference in the side effects between the two groups [157,160]. A recent review about methionine restriction in cancer treatments highlighted that not all cancers respond to methionine deprivation, and, consequently, that the identification of a predictive marker would be of great interest to select patients and cancers eligible for methionine deprivation [26].

*Methionine adenosyl transferase 2A (MAT2A) inhibitors*. MAT2A synthetizes SAM from methionine and ATP. Transient exposition of lung adenocarcinoma cells to MAT2A inhibitors completely prevented methylation, and severely decreased cell ability to form colonies, a process that was restored by the exogenous input of SAM [128]. These results indicate that transient inhibition of the methylation cycle impacts tumor growth and is an interesting therapeutic approach to further investigate. MAT2A appears as a vulnerable enzyme in cells with methylthioadenosine phosphorylase (MTAP) deletion [161], encoding another enzyme of the polyamine pathway that converts SAM to methionine and adenosine. *MTAP* and *p16/CDKN2A* are two adjacent genes located at the 9p21 locus and homozygously co-deleted in approximately 15% of all human cancers [123]. Importantly, MAT2A inhibitors have anti-proliferative activity in MTAP-deleted cancer cells and tumors [162], a result that prompted the development of a phase I clinical trial using MAT2A inhibitors (ClinicalTrials.gov NCT03435250) [163].

## 6. Remaining Questions, Hypotheses and Perspectives

### 6.1. Origin of Plasma TCI and TCII Elevation in the Context of Solid Cancer

The association between elevated transcobalamin levels and solid cancer has been well established, and reliable arguments demonstrate that the elevation of transcobalamins is secondary to the underlying cancer. However, the source of transcobalamins in the context of cancer remains unclear. High TCI and TCII levels are observed in tumor samples, which argues for an intratumoral synthesis of transcobalamins, supported by the overexpression of *TCN1* and *TCN2* in cancers cells [81,90,93,94]. However, we cannot exclude that the plasma elevation of transcobalamins is also related to cells deriving from the tumor microenvironment, notably the reticuloendothelial cells implicated in the anti-tumor immune reaction [46,164,165]. Indeed, in physiological conditions, granulocytes and endothelial cells produce most of the circulating transcobalamins [33]. However, the poor prognosis associated with plasma transcobalamins’ elevation argues more for a production by cancer cells and cancer microenvironment, rather than by anti-tumor immune cells, but it remains to identify the respective contribution to this process. Thus, a better understanding of the origin of plasma TCI and TCII in cancers will help the understanding as to which one of these markers is associated with the presence of cancer cells and which one is associated with the anti-tumoral response.

### 6.2. Plasma tB12, TCI, and TCII as Biomarkers of Solid Cancers

The association of solid cancers with plasma tB12 and TCI elevation has been well demonstrated, [13,14,15] but is only presumed for TCII [58,61]. However, studies highlighting these associations were retrospective and did not include an active cancer research strategy. Consequently, today we cannot conclude about the diagnostic performances of plasma tB12, TCI or TCII as cancer markers. Moreover, the strength of the association between tB12 elevation and solid cancers in the general population is insufficient to use this marker in a cancer screening strategy. Indeed, when used in a general population, tB12 lacks sensitivity because not all types of cancer are associated with elevated tB12 levels [13,14,15,66], and lacks specificity because other pathological conditions are associated with elevated tB12 levels, notably, myeloid blood malignancies and liver diseases [57]. This highlights the interest in specifically assessing the diagnosis performances of plasma TCI and TCII. Moreover, as the association varies according to the sites and types of cancer, a better understanding of which kinds of cancers are associated with elevated tB12/TCI/TCII levels will promote focusing on a specific population. The place of these assays in the diagnostic strategy therefore remains to be explored.

There is no consensus about the explorations to perform in the case of the fortuitous discovery of elevated B12 levels, after having eliminated the classic elevated B12-related causes, with a blood count and liver tests. This question is specifically prominent for searching for solid cancers [18,165], a situation in which explorations are expensive and induce patient anxiety. Thus, focusing on the persistent elevation of tB12 is a first decision to be made to avoid useless explorations in patients with transient elevation due to unknown acute or less specific conditions [19]. No such data exist to date for plasma TCI or TCII, but in cases of the fortuitous discovery of elevated B12 levels, it will be interesting to assess plasma TCI and TCII in order to evaluate if it is possible to improve the identification of patients with solid cancer, and to differentiate them from those with other elevated B12-related conditions. Indeed, we hypothesize that the profile of tB12/TCI/TCII elevation will differ from patients with solid cancers to patients with other elevated B12-related causes.

### 6.3. Plasma tB12, TCI and TCII as Markers for Relapses

These three markers could also be used to follow the response to treatment and to detect relapses. Indeed, previous data demonstrated an association between cancer spreading and these markers, and results from retrospective cohorts suggested their ability to detect relapses [68,69,72,73]. Thus the prospective assessment of tB12/TCI/TCII level evolutions during cancer treatment could be used on patients with an initial elevation of these markers at the time of diagnosis. Plasma TCII levels have already been prospectively demonstrated to be associated with the activity of cancers in the unique context of renal adenocarcinoma [70], but larger prospective studies including other cancer types should now investigate the ability of tB12, TCI or TCII to detect relapses in cases with elevated levels at diagnosis.

### 6.4. Plasma tB12/TCI/TCII Levels and Cancer Cell TCI/TCII/TCII-R Synthesis as Prognosis Markers

It is well established that elevated levels of plasma B12 are associated with worse prognosis in various types of cancer [16,67], but to date, the likely association between TCI and cancer prognosis is based on small retrospective cohorts [10,58]. The prognosis relevance of TCI and TCII levels should therefore be assessed to demonstrate the ability of these markers for cancer prognosis, to further establish a computational score in some types of cancer to initiate a treatment decision.

High *TCN1* expression is associated with chemotherapy resistance in colorectal cancer [74]. Other cancer types should now be investigated for this parameter, as well as for *TCN2* and *CD320* expression levels. Indeed, a prospective assessment of the association between the overexpression of *TCN1/TCN2/CD320* or high loads of TCI/TCII/TCII-R on immunohistological analyses of cancer tissues and their resistance to treatment, or for the prognosis, will help to adapt the treatment and survey strategy.

### 6.5. Plasma tB12/TCI/TCII and Cancer Cell TCI/TCII/TCII-R as Markers of a High Dependency on Cobalamin-Dependent Enzymes

We hypothesize that the plasma elevation of tB12/TCI/TCII levels in cancer, or cancer cell overexpression of TCI/TCII/TCII-R, will demonstrate a metabolic singularity linked to a high dependency to cobalamin and cobalamin-related enzymes [33,47]. The same reasoning was applied to elevated plasma and tumor TCI/TCII levels in some cancers reflecting higher B12 needs [93]. This will explain why not all cancers are associated with TCI/TCII/TCII-R plasma and tumor singularities, as dependency to cobalamin-dependent enzymes and notably to methionine synthase, and consequently on exogenous methionine, vary among the different cancer types [121,124,125]. This was supported by the demonstrated increased TCII-R expression in cancer cells under methionine deprivation [91].

**Figure 4:**
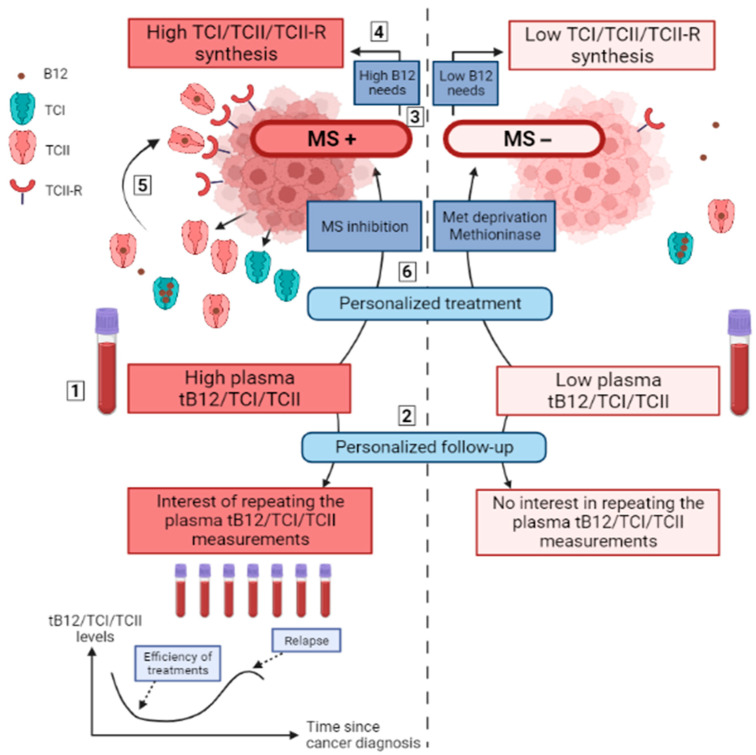
Remaining questions and hypotheses about the relationships between cobalamin-related metabolic pathways and plasma tB12/TCI/TCII measurements in solid cancers. Notes: Elevated plasma tB12, TCI and TCII levels are associated with the diagnosis of solid cancer, but their exact diagnostic performances remain to be assessed, as well as the explorations to perform when searching for cancer in cases of the fortuitous discovery of high tB12 (1). Plasma levels of tB12, TCI and TCII are associated with the course of cancers and could be useful to detect relapses early in the case of a previous elevation at diagnosis, whereas these measurements may be useless in cases of initial normal levels (2). It is demonstrated that cancer cells may be classified according to the activity of the methionine synthase and their dependency on exogenous methionine (3). A hypothesis proposes that the synthesis of TCI, TCII and TCII-R from cancer cells is associated to an elevated activity of MS or MMCoAMut with high B12 needs (4). The synthesis of TCI, TCII and TCII-R from cancer cells favors high B12 uptake (5) and leads to elevated plasma tB12, TCI and TCII levels (1). Plasma measurements of tB12, TCI and TCII could therefore indicate the B12 needs of cancer cells to support the activity of MS and MMCoAMut, which could help determine the most appropriate treatment: the inhibition of MS in the case of high MS activity; methionine deprivation in the case of low MS activity with high exogenous methionine needs (6). B12: vitamin B12 (cobalamin); MMCoAMut: methylmalonyl-CoenzymeA mutase; MS: methionine synthase; MS+: high activity of methionine synthase (independent from exogenous methionine); MS−: low activity of methionine synthase (dependent from exogenous methionine). tB12: total plasma vitamin B12; TCI: transcobalamin I (haptocorrin); TCII: transcobalamin II; TCII-R: transcobalamin II receptor. Figure created with BioRender.com accessed on 25 April 2022.

Consequently, TCI and TCII secretions, together with TCII-R expression could be relevant markers of a metabolic singularity of some cancers with higher methionine dependency, rather than being general markers of all cancers (Figure 4). Such a correlation might also exist for the requirement of succinyl-CoA, but its association with MMCoAMut activity related to cobalamin remains less investigated than that of methionine synthase in cancer development.

If former hypotheses are confirmed, important therapeutic outcomes will arise because the TCI, TCII and TCII-R markers will be useful to differentiate cancers with high methionine synthase activity from cancers with a high need for exogenous methionine, which are likely to better respond to the inhibition of methionine synthase or to methionine-depletive therapies, respectively. Methionine-depletion has already provided significant benefits in many studies, but rather poor benefits when applied to unscreened cancers [26]. Thus, these treatments will have significant efficiency in methionine-dependent cancers when these biomarkers are identified, allowing the screening of cancers sensitive to methionine deprivation. Altogether, to summarize these hypotheses, we propose that plasma and tumor TCI/TCII/TCII-R levels might provide these relevant biomarkers and should, prospectively, be assessed in many solid cancers.

## 7. Conclusions

Cobalamin has a role in many key pathways required for cancer cells’ development: methylation through methionine synthase, succinylation through MMCoAMut, purine base synthesis and ATP production. It was therefore expected that pathological links exist between solid cancers and cobalamin metabolism. Effectively, the elevation of plasma tB12/TCI/TCII levels are associated with cancer diagnosis and worse prognosis, with levels paralleling the cancer course during and after treatment. In addition, high levels of TCI/TCII/TCII-R synthesis in cancer cells are associated with worse prognosis and higher resistance to chemotherapy. However, many questions remain unanswered and deserve further studies: (i) to situate plasma B12/TCI/TCII measurements in cancer diagnosis, prognosis and relapse detection strategies, (ii) to include TCI/TCII plasma measurements and TCI/TCII/TCII-R cancer expressions in the therapeutic and survey strategies, and (iii) to investigate the links between TCI/TCII/TCII-R synthesis and the metabolic dependency to cobalamin-related pathways. The results of these studies will ensure better diagnosis and follow-up of patients, together with the opening of therapeutic routes to treat solid cancer.

## Abbreviations

ASGPR: asialoglycoprotein receptor; ATP: adenosine triphosphate; B12: vitamin B12 (cobalamin); CBS: cystathionine beta synthase; CRP: C-reactive protein; CSC: cancer stem cell; EMT: epithelial–mesenchymal transition; MAT2A: methionine adenosyl transferase 2A; MMCoAMut: methylmalonyl-CoenzymeA mutase; MTAP: methylthioadenosine phosphorylase. NO: nitric oxide; OR: odds ratio; ROS: reactive oxygen species; SAH: S-adenosylhomocysteine; SAM: S-adenosylmethionine; tB12: total plasma vitamin B12; SNP: single nucleotide polymorphism; TCI: transcobalamin I (haptocorrin); TCII: transcobalamin II; TCII-R: transcobalamin II receptor; TCA cycle: tricarboxylic acid Krebs cycle.
